# HIIT and Tabata protocols for improving physical and cognitive health in sedentary college students: a randomized trial

**DOI:** 10.3389/fpsyg.2026.1656208

**Published:** 2026-02-26

**Authors:** Yadong Xue, Ning Xu, Meng Zhang

**Affiliations:** 1 School of Physical Education, Yan'an University, Yan'an, Shaanxi, China; 2 School of Physical Education, Guangzhou Huali College, Guangzhou, China; 3 School of Exercise and Health, Shanghai University of Sport, Shanghai, China

**Keywords:** cognitive function enhancement, high-intensity interval training, physical fitness, sedentary college students, Tabata protocol

## Abstract

**Introduction:**

This randomized controlled trial aimed to compare the effects of HIIT-30S protocols (30 s work/30 s rest) and Tabata protocols (20 s work/10 s rest) protocols on physical fitness and cognitive function in sedentary college students.

**Methods:**

Eighty-four undergraduates (19.07 ± 0.76 years; 34 males and 60 females) were stratified into HIIT-30S, Tabata, or moderate-intensity training (MICT) groups and completed 24 supervised sessions over 8 weeks. Physical outcomes included an 800-m run (cardiorespiratory endurance), push-ups/sit-ups (muscular endurance), 50-m sprint (speed), the Sitting Reach Test (flexibility), and heart rate recovery (cardiovascular recovery ability). Cognitive assessments encompassed the WAIS-IV core cognition (general intelligence), letter-number sequencing test (working memory), time management questionnaire (TMQ), emotion regulation questionnaire (ERQ), reaction time testing, and a 12 weeks follow-up comprehensive cognition questionnaire. Statistical significance was set at *P* < 0.05.

**Results:**

HIIT-30S and Tabata outperformed MICT in all domains (*P* < 0.05). HIIT-30S elicited superior physical adaptations: 800-m run time decreased by −11.78 s, push-ups increased +9.29 reps, sit-ups increased +11.39 reps, 50-m sprint decreased −0.37 s, and HRR improved +12.66%. Tabata showed greater neurocognitive enhancements: WAIS-IV core cognition scores rose +10.47 points, letter-number sequencing scores rose +1.68 points, TMA scores rose +10.97 points, EMA scores rose +16.79 points, simple reaction time decreased −40.61 ms, choice reaction time decreased −65.15 ms, and Continuous Reaction time decreased −51.14 ms. At the 12-week follow-up, HIIT-30S maintained cognitive gains (+3.05%) with the greatest improvement compared to Tabata (+1.97%).

**Discussion:**

These findings indicate that the work-to-rest ratio is a key determinant of training effects, enabling protocol customization for specific individual fitness and cognitive aims.

## 1 Introduction

During adolescence, reduced physical activity, irregular sleep, and increasing self-regulatory demands can negatively impact the physical and mental health of young adults (Sheldon et al., 2021; Pinto et al., 2024; Martínez-Riera et al., 2018). Epidemiological data reveal alarming trends among college populations: over 60% report sleep disturbances (Gardani et al., 2022), 45% engage in sedentary behaviors exceeding 8 h daily (Zajac et al., 2020), and 30% develop maladaptive dietary habits (Cleary et al., 2011; Cheikh Ismail et al., 2024; Janssen et al., 2018). A recent study showed that the physical fitness of male students decreased significantly 1 year after entering college, and this trend was also observed among students who enjoyed sports (Dong et al., 2023; O'Brien et al., 2022). Over 4 years of college, the average weight gain is 4.5 kg for males and 5.4 kg for females (Wetter et al., 2013). Weight gain not only increases the risk of chronic diseases but also aggravates mental health issues, including depression and anxiety (Sheldon et al., 2021; Cleary et al., 2011; Ahmed et al., 2023). Severe mental health problems can lead to cognitive decline, especially affecting attention and reaction speed (Putri and Imran, 2022; Adaili et al., 2016).

High-Intensity Interval Training (HIIT) has emerged as a promising intervention for improving neurological and cognitive functions, including attention and memory (Gomez-Pinilla and Hillman, 2013; Reyes-Amigo et al., 2022). HIIT, characterized by short bursts of intense exercise at ≥85% of maximum heart rate (HRmax) through BDNF-mediated synaptic plasticity and increased cerebral oxygenation, enhance cardiovascular endurance, neurotransmitter release, and cognitive function (Mosley, 2014; Ballester-Ferrer et al., 2022). High-intensity interval training can significantly improve executive functioning in children and adolescents (Reyes-Amigo et al., 2022). HIIT at different intensities and durations produces varying training effects in college athletes. A training-to-rest ratio of 1:4 is more effective in promoting the development of cardiopulmonary function compared to ratios of 1:2 or 1:8 (Seo et al., 2019). Training at a high intensity of 95% is more effective than training at 85% in improving the physical fitness and reaction speed of handball players (Viaño-Santasmarinas et al., 2018). Moreover, HIIT has been shown to significantly improve cardiovascular health, muscle strength, metabolic efficiency, and overall body composition in adolescents, while also enhancing cerebral blood flow and neural conduction (Ballester-Ferrer et al., 2022; Liu and Li, 2024; Plizga et al., 2024). The Tabata Training protocol, a form of HIIT, involves 20 s of maximum effort followed by 10 s of rest and has been recognized for its ability to enhance cardiovascular and muscular fitness within a concise 4-min session (Olson, 2014). Its structured design is particularly beneficial for novices, as it facilitates rapid adaptation and progressive improvements (Tabata, 2019). Studies have demonstrated its effectiveness in improving aerobic capacity and muscular strength and addressing the time constraints faced by students (Lu, 2023).

Although numerous studies have suggested that HIIT can improve fitness and cognitive ability in teenagers (Ballester-Ferrer et al., 2022; Seo et al., 2019; Liu and Li, 2024; Shao et al., 2023a), personalized HIIT intervention programs tailored to the unique temporal and spatial constraints (such as busy academic schedules and limited exercise time) and motivational characteristics (such as being beginners and lack of exercise habits) of sedentary university students are still relatively scarce. This limits the effective promotion and application of HIIT in university students. Meanwhile, the efficacy of HIIT in enhancing physical fitness is influenced by factors such as training session duration, interval length, exercise intensity, workout structure, and number of sets (Mosley, 2014). Variations in these parameters may yield different training effects (Shao et al., 2023a; Wang et al., 2023), whereas inappropriate prescription carries the risk of overtraining with potentially detrimental effects on cognitive function (Plizga et al., 2024).

Targeting sedentary university students characterized by low baseline fitness, weight gain, limited exercise experience, low intrinsic motivation, and time constraints, this study implemented two iso-temporal HIIT protocols: (1) Tabata protocol (20 s work: 10 s rest); and (2) HIIT-30S protocol (30 s work: 30 s rest). Both protocols primarily utilized bodyweight exercises (e.g., push-ups, abdominal crunches, planks), with training intensity progressively increased from ≥65 to ≥85% of age-predicted maximal heart rate. The primary objectives were as follows: (1) to enhance cognitive function in sedentary college students through structured programming; (2) to improve cardiorespiratory fitness and reduce cardiovascular disease risk via 20–30 s high-intensity stimuli; and (3) to augment baseline physical fitness and reduce body fat percentage. Therefore, this study aimed to comparatively examine the differential effects of these protocols on physical fitness, cognitive function, and program adherence in sedentary university students. We hypothesized that (1) both protocols would significantly improve physical fitness and cognitive function, with the HIIT-30S protocol yielding greater physical fitness enhancements due to the longer high-intensity stimulus duration; and (2) the strictly defined intervals and inherent motivational aspects of the Tabata protocol may enhance adherence among participants with lower baseline intrinsic motivation levels. These findings are anticipated to establish evidence-based guidelines and provide a reference framework for university students engaged in physical exercise to improve their physical and cognitive health.

## 2 Methods

### 2.1 Experimental approach to the problem

**Figure 1:**
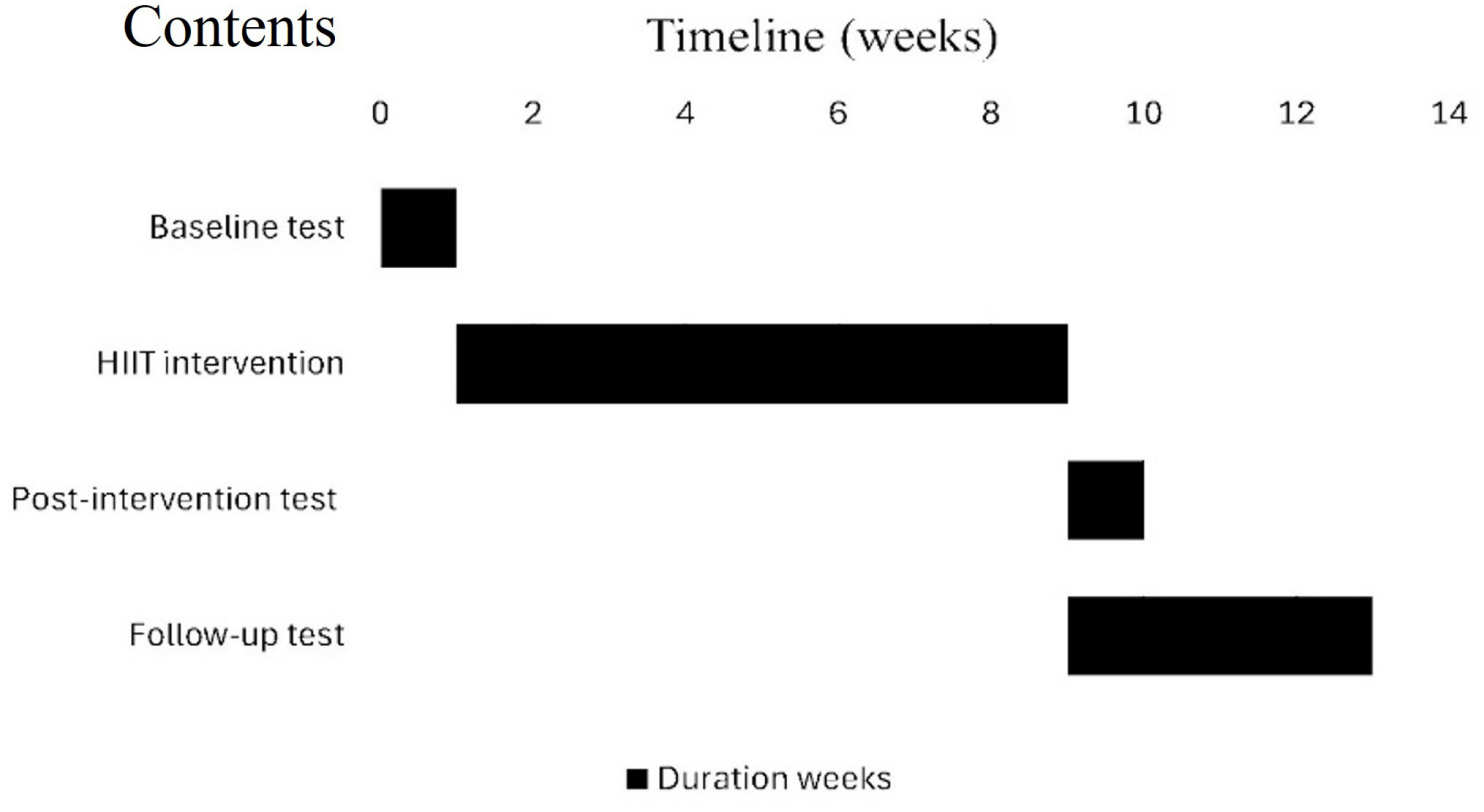
Experimental timeline: baseline assessments (physical fitness and cognitive tests) were conducted 1 week prior to the 8-week intervention period. Participants completed 24 supervised sessions (3 sessions/week) of HIIT-30S (30 s:30 s work-rest ratio at ≥85% HRmax), Tabata (20 s:10 s work-rest ratio at ≥90% HRmax), or MICT (continuous training at 65% HRmax). Follow-up cognitive assessments were conducted at 8 weeks (post-intervention) and 12 weeks (retention).

This randomized trial explored the effects of two HIIT protocols on the physical fitness and cognitive function of sedentary college students. Specifically targeting sedentary college students with low basic physical fitness, weight gain, limited exercise experience, low intrinsic motivation, and high life and academic pressure, we designed two isochronous HIIT protocols. Leveraging HIIT's time-efficient characteristics of HIIT to elicit rapid physiological adaptations, these protocols aim to enhance participant engagement through their high-efficacy and time-sparing characteristics. High-intensity stimuli of 20–30 s were implemented to improve the participants' physical fitness and cognitive abilities. After baseline assessments, participants were randomly assigned to the HIIT-30S, Tabata, or Control group via stratified randomization using SPSS (version 26.0; IBM Corp., Armonk, NY, USA). Stratification was based on age, baseline physical fitness, and cognitive ability measured during the baseline evaluation (Table 1). The researchers adhered to a blinded allocation process for the study. This method aims to balance the key characteristics among groups and minimize potential biases inherent in the randomization process. The HIIT-30S protocol involved four sets per training unit performed at a 30:30 s work-to-rest ratio, whereas the Tabata protocol involved eight sets per training unit at a 20:10 s work-to-rest ratio. The control group (CG) completed 4 min of moderate-intensity continuous training (MIT) per training unit, with 1–3 min of rest between each unit. All three protocols were structured around two 4-min training units, separated by 1–3 min of rest. Training was conducted three times a week for 8 weeks, totaling 24 sessions, each including a warm-up, HIIT protocol, and relaxation phase. The duration of each phase was consistent across all three groups. Figure 1 presents a detailed timeline illustrating the progress of the study.

**Table 1:** Baseline test results for subjects (mean ± standard deviation) and one-way ANOVA results.

**Tests**	**HIIT (*N* = 28)**	**Tabata (*N* = 28)**	**MICT (*N* = 28)**	***P*-value**
Age (year)	18.93 ± 0.66	19.07 ± 0.72	19.21 ± 0.88	0.373
Weight (kg)	65.04 ± 11.16	64.75 ± 10.43	65.79 ± 11.46	0.936
BMI (kg/m^2^)	22.36 ± 2.41	22.59 ± 2.17	22.88 ± 2.40	0.707
**Physical fitness**				
Push-ups	21.89 ± 9.50	21.64 ± 8.28	22.14 ± 7.04	0.975
Sit-ups	34.11 ± 3.35	33.32 ± 3.13	32.86 ± 3.04	0.335
50 m (s)	8.47 ± 0.76	8.38 ± 0.57	8.53 ± 0.64	0.714
Sitting reach test (cm)	18.03 ± 5.12	18.76 ± 5.44	18.1 ± 5.15	0.849
800 m (s)	219.21 ± 22.22	217.71 ± 19.54	218.32 ± 18.90	0.962
Heart rate reserve (%)	2.54 ± 0.27	2.61 ± 0.26	2.6 ± 0.24	0.528
**Cognitive ability**				
Core cognitive ability	106.93 ± 2.53	107.23 ± 2.98	108.59 ± 2.63	0.129
Letter-number sequencing	8.90 ± 0.89	8.93 ± 0.85	9.0 ± 0.79	0.914
Time management assessment	66.43 ± 3.96	67.14 ± 4.04	66.86 ± 3.18	0.773
Emotional regulation ability	66.96 ± 4.58	66.07 ± 4.97	64.46 ± 4.38	0.132
Simple reaction time	249.11 ± 18.98	254.57 ± 18.37	247.75 ± 18.58	0.335
Choose reaction time	377.54 ± 24.24	390.86 ± 29.41	382.64 ± 21.91	0.147
Continuous reaction time	493.96 ± 30.65	501.07 ± 27.93	495.54 ± 18.07	0.567

**Figure 2:**
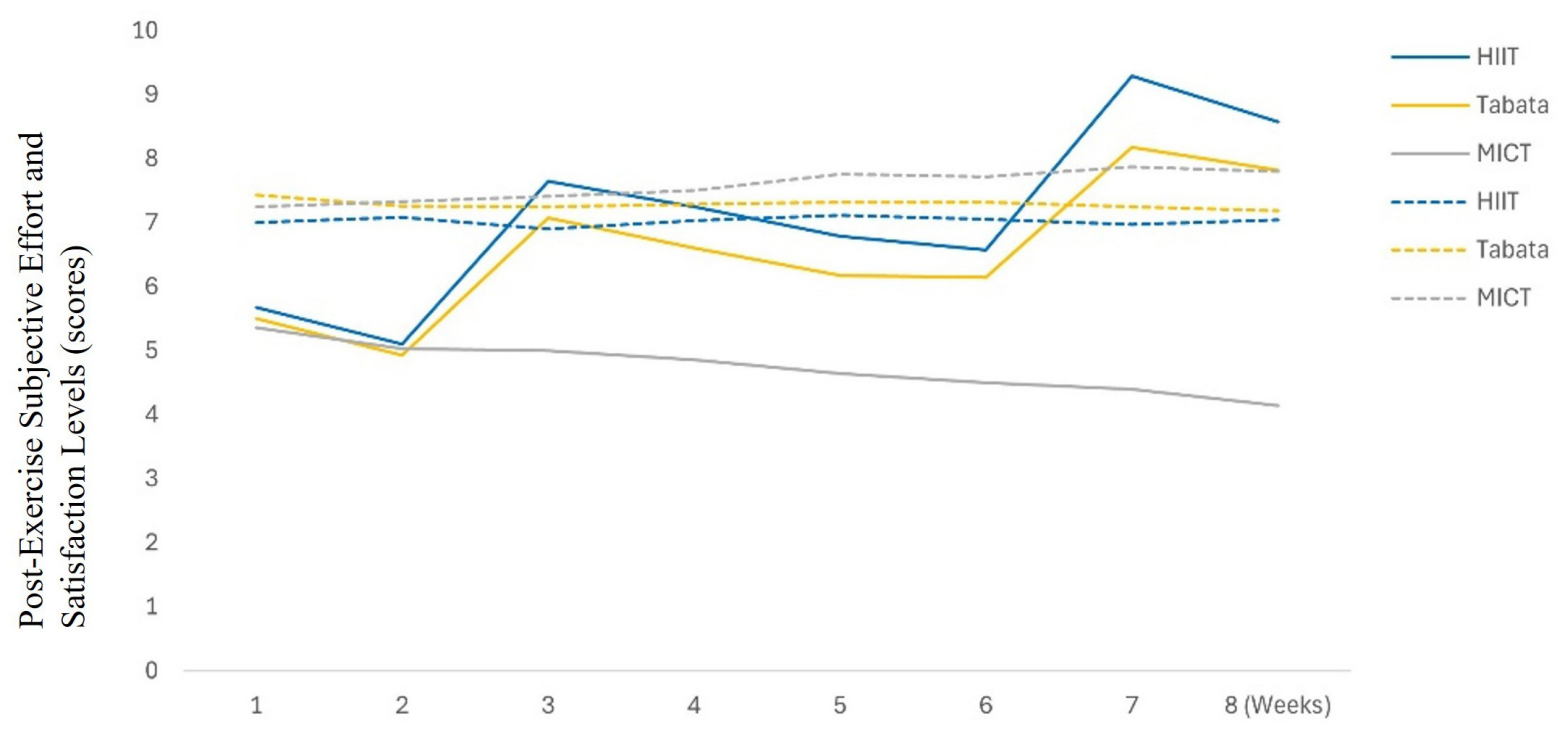
Longitudinal changes in comprehensive cognition questionnaire. Time course of composite cognitive scores (baseline, 8-week post-intervention, 12-week follow-up). HIIT-30S maintained superior gains at follow-up (+3.05% vs. Tabata: +1.97%, MICT: +1.46%).

**Figure 3:**
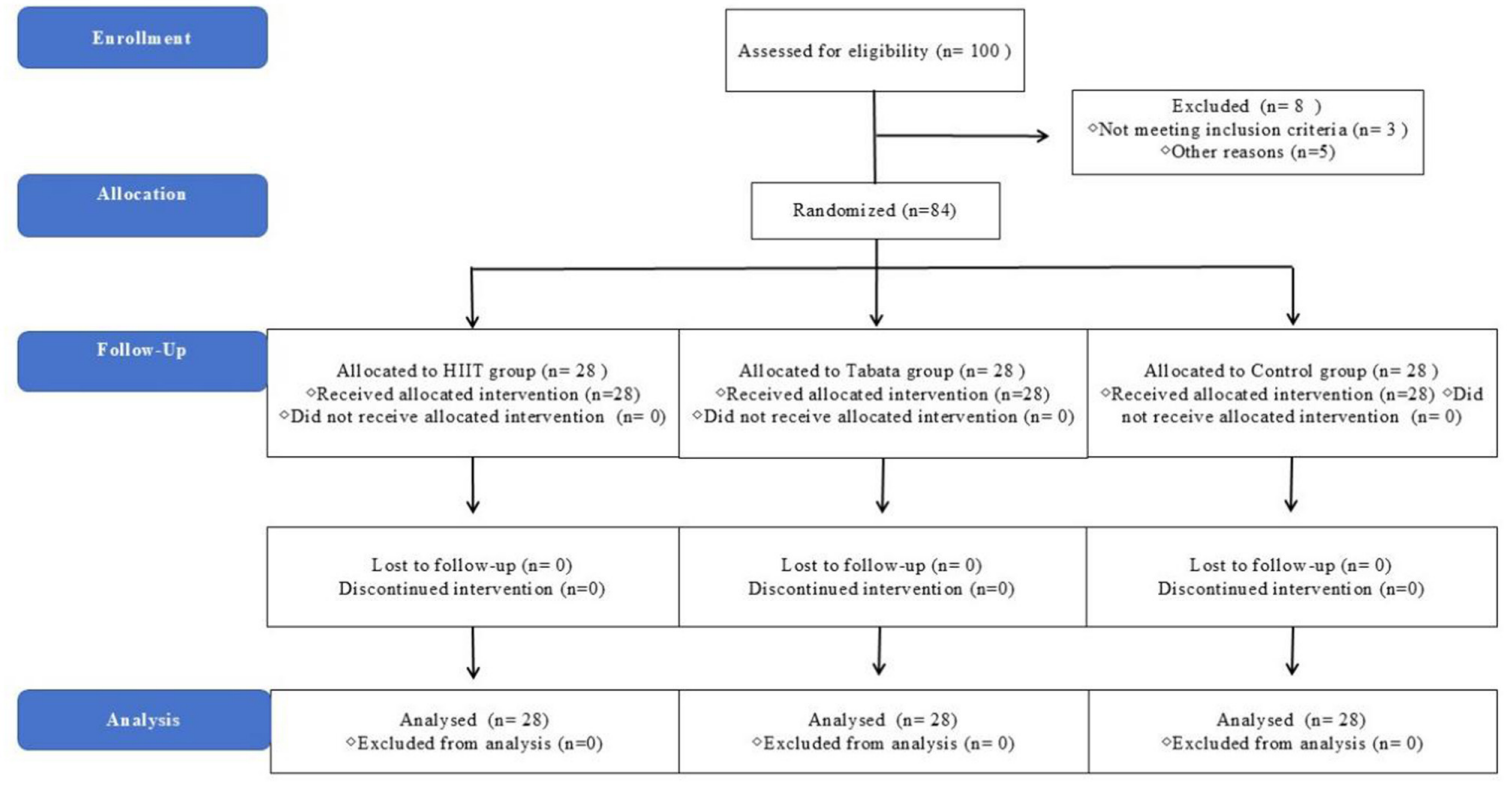
CONSORT diagram of randomized controlled trial. Flowchart illustrating participant enrollment, randomization, and retention throughout the 8-week intervention. A total of 100 college students were screened, with 84 meeting inclusion criteria and randomly allocated to HIIT-30S (30 s work/30 s rest, *n* = 28), Tabata (20 s work/10 s rest, *n* = 28), or MICT (65% HRmax, *n* = 28) groups. No participants discontinued due to schedule conflicts or personal reasons. Final analyses included 28 completers per group.

Based on the “National Physical Fitness Testing Program for College Students” and the training content, a physical fitness testing protocol was designed to accommodate the participants' specific characteristics. This protocol included the following tests: 1-min push-ups to assess upper limb endurance, sit-ups to assess core strength, an 800-m run to assess cardiorespiratory endurance, a 50-m sprint to assess lower limb explosive strength and speed, and a sitting forward bend test to assess flexibility. Core cognitive abilities were assessed using WAIS-IV Language and Performance composite scores. The letter-number sequencing test evaluated memory and processing speed, and the reaction time test assessed processing speed and executive function. Additionally, self-report questionnaires measuring time management and emotion regulation were used to assess executive functioning and self-regulatory abilities, respectively. To track changes in cognitive function, a bespoke comprehensive cognition questionnaire, which was designed to correspond to the physical, psychological, and environmental characteristics of college students, was administered over a period of 13 weeks, from 1 week before the initiation of the intervention to 12 weeks following its conclusion (Figure 2) with a Cronbach's alpha coefficient for CCQ was 0.81. The assessors were blinded to the group allocation to maintain the integrity of the study. Figure 3 shous the experimental procedure. To protect the participants' confidentiality, all questionnaires were anonymized using the HLxx codes. Compliance with the training protocols was strictly monitored through daily logs and regular interactions with the research team.

### 2.2 Participants

Participants were recruited via campus media and informational posters at Guangzhou HuaLi University, resulting in the enrollment of 100 undergraduate students. This is in accordance with the findings of Wang et al. (2023). *A priori* power analysis was conducted using G^*^Power 3.1 (Faul et al., 2007). The analysis targeted 80% power (α = 0.05, β = 0.20) to detect a medium effect size (*F* = 0.25, $\eta_{p}^{2}$ = 0.06) for time × group interactions in repeated-measures ANOVA, assuming a conservative correlation of *r* = 0.60 among repeated measurements based on pilot data from our laboratory. This calculation indicated a minimum requirement of seventy-two participants. To account for potential attrition and enhance statistical precision, we increased the sample size to 28 participants per group (achieved power = 80%). Following the initial screening, 16 participants were excluded from the study due to personal circumstances or medical contraindications, resulting in a final sample size of 84 (34 males and 60 females; Figure 1). The inclusion criteria were as follows: (1) age between 18 and 22 years; (2) absence of injuries or medical conditions precluding high-intensity activity; and (3) non-engagement in high-intensity physical activities within the last 3 months, with a weekly exercise duration of less than 3 h. The research protocol was reviewed and approved by the Ethics Committee of Guangzhou HuaLi University in accordance with the principles of the Helsinki Declaration to ensure adherence to ethical standards and protection of participant rights. Informed consent was obtained from all participants in writing, encompassing a comprehensive disclosure of the study's objectives, procedures, potential risks, and anticipated benefits.

### 2.3 Training protocol

**Figure 4:**
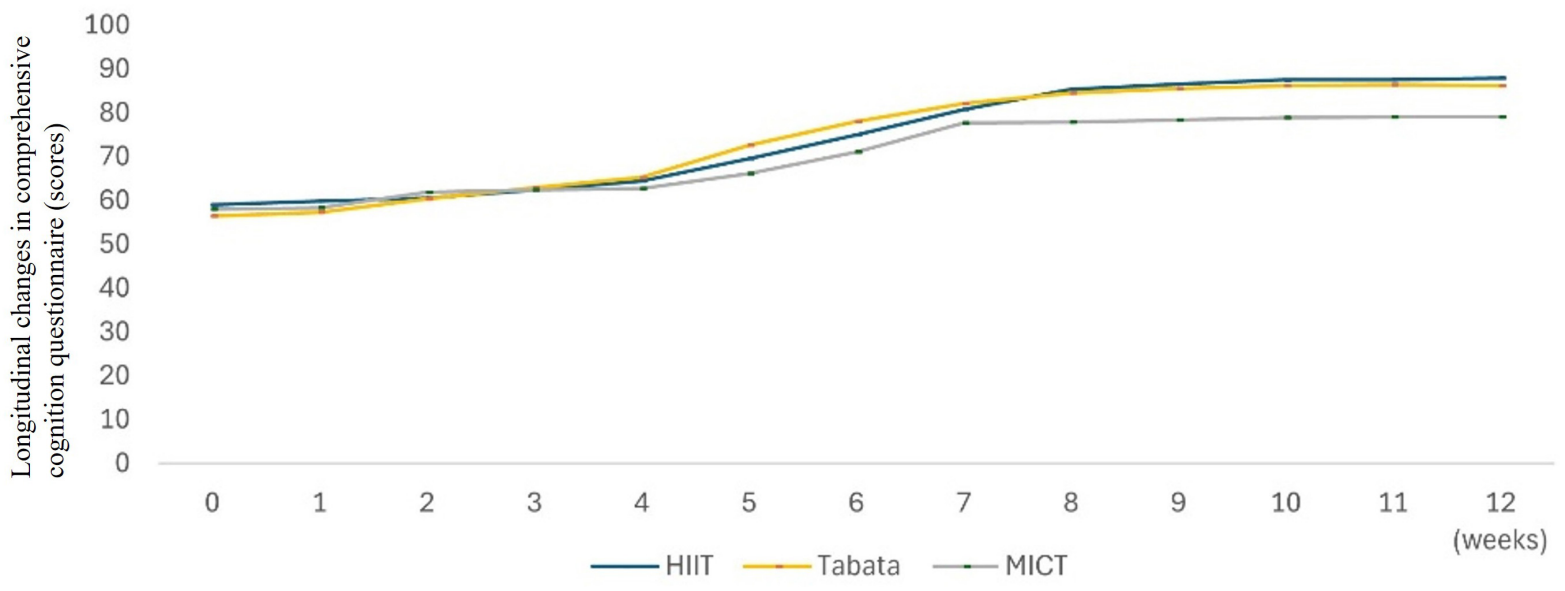
The subjective effort level post-exercise and satisfaction levels. Subjective rating of perceived exertion (RPE) statistics (solid line), and subject satisfaction and acceptability post-training statistics (dashed line).

Training was conducted three times a week for 8 weeks (24 sessions). Each session followed a standardized structure, beginning with a 10-min warm-up of low-intensity aerobic exercise (e.g., brisk walking on a treadmill or cycling). This was followed by the core HIIT training period and concluded with a 10-min stretching and relaxation routine to promote recovery and improve flexibility. The core HIIT training period lasted 9–11 min and always involved two 4-min training units, performed back-to-back, with 1–3 min of rest recovery between the units. During the work intervals, the participants performed high-intensity bodyweight resistance exercises (e.g., burpees, planks, bodyweight squats, and pushups) with maximal effort. The intensity for all HIIT sessions was rigorously controlled and maintained within the target heart rate range (Table 2) calculated using the Karvonen method (Karvonen and Vuorimaa, 1988). The HIIT-30S protocol involved four sets of intervals per training unit at a 30:30 s work-to-rest ratio, whereas the Tabata protocol involved eight sets of intervals per training unit at a 20:10 s work-to-rest ratio. The Control Group (CG) completed two training units of 4-min moderate-intensity continuous training with 1–3 min rest between units. Notably, the duration of the warm-up period, structure of the core HIIT/MICT training period (two 4-min work units with inter-unit rest), and relaxation period were identical across all three groups throughout the study. The heart rate was continuously monitored using a Huawei Watch 4 Pro during the work intervals of each session. Perceived exertion levels were evaluated using the Rating of Perceived Exertion (RPE) scale (10-point version) immediately after completing each session. Additionally, participants' satisfaction and preferences concerning the assigned training protocol were collected to provide feedback for potential future modifications (Figure 4). To maintain the integrity of the study, the participants were instructed to strictly adhere to their assigned training protocols throughout the study duration. Furthermore, they were advised to limit their intake of high-calorie foods, avoid alcohol consumption, and maintain adequate sleep (e.g., 7–9 h per night) outside the training sessions.

**Table 2:** Details of training protocols for 8 weeks.

**Period**	**Group**	**Training protocols**
1–2 weeks	HIIT (*N* = 28)	Training content: JR, LL, SSL, PU. 4 × [4 × (30:30 s)/3 min recovery]; intensity ≥ HRmax 65–75%.
	Tabata (*N* = 28)	Training content: JR, HR, LU, AF, SC, SSL, PU, KP. 4 × [8 × (20:10 s)/3 min recovery]; intensity ≥ HRmax 65–75%.
	MICT (*N* = 28)	Training content: JR, HR, LU, AF, SC, SSL, PU, KP. 4 × [(1 × 4 min)/3 min recovery]; intensity ≥ HRmax 40–50%.
3–6 weeks	HIIT (*N* = 28)	Training content: JJ, SLS, STC, PU. 4 × [4 × (30:30 s)/2 min recovery]; intensity ≥ HRmax 75–85%.
	Tabata (*N* = 28)	Training content: JJ, SLH, SLS, SAT, STC, PU, BJ, HKR. 4 × [8 × (20:10 s)/2 min recovery]; intensity ≥ HRmax 80–90%.
	MICT (*N* = 28)	Training content: JJ, SLH, SLS, SAT, STC, PU, BJ, HKR. 4 × [(1 × 4 min)/2 min recovery]; intensity ≥ HRmax 55–65%.
7–8 weeks	HIIT (*N* = 28)	Training content: HKR, Squate, Plank, BJ. 4 × [4 × (30:30 s)/1 min recovery]; intensity ≥ HRmax 85%.
	Tabata (*N* = 28)	Training content: RF, HKR, HSJ, Squate, Plank, SP, BJ. 4 × [8 × (20:10 s)/1 min recovery]; intensity ≥ HRmax 90%.
	MICT (*N* = 28)	Training content: JJ, LU, AF, SLH, HSJ, SSL, SC, Squate. 4 × [(1 × 4 min)/1 min recovery]; intensity ≥ HRmax 65%.

4 × [4 × (30:30 s) 3 min/2 min/1 min recovery]: 4 rounds of 4 min training and 3/2/1 min recovery, 4 sets of 30:30 s HIIT per round; 4 × [8 × (20:10 s) 3 min/2 min/1 min recovery]: 4 rounds of 4 min training and 3/2/1 min recovery, 8 sets of 20:10 s Tabata training per round; 4 × [(1 × 4 min)/3/2/1 min recovery]: 4 rounds of 4 min training and 3/2/1 min recovery, 4 min MICT per round, No rest time.

HRmax, maximum heart rate; JR, jump rope; LL, lateral lunge; SSL, supine scissors legs; PU, push-ups; KP, kneeling push-ups; HR, heel raising; LU, leg up and high five; AF, alternating front leg kicks; SC, supine curl-ups; JJ, jumping jacks; SLS, side lunge squats; STC, supine trunk curlsl; SLH, single-leg hops (alternating sides); HSJ, half-squat jumps; SAT, supine alternating touch-downs; HKR, high-knee running; BJ, burpee jump; RF, running fast in place; HSJ, half squat jump; SP, side plank (left and right).

### 2.4 Procedures

The experimental phase was conducted in a standardized and controlled environment under the supervision of a research team. Rigorous training of both participants and staff ensured safety, minimized bias, and maintained robust results. The standardized physical fitness assessment began with a 15-min moderate jog, followed by exercises including half push-ups, rope jumping, a 30-m sprint, and comprehensive stretching to activate the musculoskeletal system. Cognitive assessments were timed to occur between the physical test components with planned rest intervals to maintain optimal participant performance. Strict adherence to the inclusion criteria was enforced, requiring participants to (1) have sufficient sleep the night before testing; (2) abstain from alcohol and caffeine for at least 3 h prior; (3) avoid heavy meals within 3 h; and (4) be free from recent physical injuries.

#### 2.4.1 Cognitive ability assessment

##### 2.4.1.1 Core cognitive function test

Participants were evaluated using the WASI-IV Chinese Version in a controlled, illuminated setting following operational protocols (Wang et al., 2015). The Language (verbal skills) and Performance (spatial skills and problem-solving) Ability subtests were administered sequentially according to the WAIS-IV manual and the time guidelines. The raw scores were transformed into T-scores and averaged to determine the overall cognitive score. The participants completed two independent assessments, each separated by a 20-min rest period.

##### 2.4.1.2 Letter-number sequencing test

The Chinese version of the WAIS-IV letter-number sequencing subtest was used to assess working memory abilities (Wang et al., 2015). During the test administration, the participants were instructed to immediately replicate the sequence of letters and numbers provided by the examiner. For each sequence, the examiner recorded the accuracy of the responses and the number of prompts required. Raw scores were transformed into T-scores and combined with those from other cognitive tests to calculate the overall cognitive composite score. To prevent participant fatigue and maintain performance at peak levels, a 20-min break was interspersed between the two independent assessments; ICC was 0.90 (Mielicki et al., 2018) (95% CI: 0.870–0.993).

##### 2.4.1.3 Time management ability

Participants' time management ability was evaluated using the college student time management questionnaire (TMQ). The TMQ, developed from literature and expert reviews, covers planning, prioritization, and efficiency. Responses were rated on a five-point Likert scale with a Cronbach's alpha coefficient for TMQ was 0.87 (Alay and Kocak, 2002).

##### 2.4.1.4 Reaction time testing

Using E-Prime (Chinese 2.0 Version), participants underwent three reaction time tests: simple reaction time (SRT; where they pressed “1” upon its appearance, ICC = 0.918, SEM = 0.05 s), Choice Reaction time (CRT; involving responding to digits like “1” or “2” with increasing difficulty, ICC = 0.832, SEM = 0.05 s), and Continuous Reaction time (CRT; testing reactions to digit sequences, ICC = 0.756, SEM = 0.05 s). The stimulus rates were constant for all tests, which include multiple trials with short breaks. All tests were conducted in a controlled, illuminated setting, following the operational protocols.

##### 2.4.1.5 Emotional regulation ability

To evaluate the emotional regulation abilities of college students, we drew upon cutting-edge theories and Cheng-Hsien Li's research findings (Li and Wu, 2020). Tailoring the assessment to the psychological characteristics of college students, we developed the “Emotional Regulation Questionnaire for Contemporary Chinese College Students” (ERQ). The ERQ consists of 20 items rated on a five-point Likert scale designed to evaluate cognitive-related emotion regulation. The participants were fully briefed on the study, including instructions and privacy guarantees. The data were collected online, encrypted, and anonymized prior to analysis. Two ERQ assessments were performed 20 min apart. The internal consistency Cronbach's α was 0.71–0.85 (Zhao et al., 2020).

#### 2.4.2 Physical fitness assessment

##### 2.4.2.1 Push-up test

The One-Minute Push-Up Test was conducted to assess upper body strength and endurance (Artanayasa et al., 2023). The participants performed push-ups for 1 min, which were monitored by two independent testers using stopwatches. Each participant completed two trials with 3–5 min of rest in between, and the highest count was recorded. The ICC for these trials was 0.94 (Rubin et al., 2025) (95% CI, 0.777–0.943).

##### 2.4.2.2 50-m sprint test

Lower limb explosive power and sprint speed were measured over a 50-m sprint on a flat track (Qu et al., 2021). Three staff members timed the participants, and each participant completed three trials, with the highest score recorded. Each test was administered with a rest period of 3–5 min between attempts to ensure the participants' physical recovery. The ICC for these trials was 0.96 (Barbosa et al., 2020) (CI: 0.854–0.974).

##### 2.4.2.3 One-minute sit-up test

This study assessed abdominal and lumbar muscle strength and endurance using a one-minute sit-up test (Diener et al., 1995). The participants lifted their upper bodies using their abdominal muscles until their scapulae contacted the ground or a line. Two trained testers timed and counted the number of sit-ups, with three trials and a 3–5 min rest between each test. The highest score was obtained for the final measure; ICC was 0.83 (Seger et al., 2022) (CI: 0.352–0.852).

##### 2.4.2.4 Sitting reach test

Flexibility was evaluated using a standardized SIT with a 1.5-m board. The participants sat with their legs straight, flat, and bent forward to touch the ground with their fingertips. Three trials with 3–5 min rest were conducted, and the highest score was recorded, with an ICC of 0.92 (Ayala et al., 2012) (CI: 0.67–0.938).

##### 2.4.2.5 The 800-m run test and HRR

The study utilized an 800-m running test on a 400-m track to assess cardiorespiratory fitness (Li, 2022). Participants started from a stationary position at the start line and ran a 800-m distance at their maximum possible speed. Upon completion of the 800-m test, real-time heart rate data were collected using a Huawei Watch 4 smartwatch and the Huawei Health app. The heart rate at 60 s post-exercise was also recorded. The heart Rate Recovery (HRR) was calculated using a standard formula.


$$\begin{array}{r} Heart\;rate\;recovery\;rate\;\left(HRR\right)\\ =\frac{HRmax\;-\;Heart\;rate\;at\;60\;s}{10}\times 100\% \end{array}$$ (1)

### 2.5 Statistical analyses

Data analysis was conducted using SPSS Statistics (version 26.0; IBM Corp., Armonk, NY, USA), and normality was evaluated using the Shapiro-Wilk test. Homogeneity of variance was assessed using Levene's test. Reliability was determined using the Intraclass Correlation Coefficient (ICC) with single measures, interpreting values < 0.4 as poor, 0.4–0.74 as moderate, and ≥0.75 as excellent reliability (Koo and Li, 2016). One-way ANOVA was used to assess baseline differences among the HIIT-30S, Tabata, and control groups for the primary outcome measures (e.g., BMI, Push-ups and Core cognitive ability). A repeated-measures two-way ANOVA (group × time) was used to analyze pre- and post-intervention data. Sphericity was tested using Mauchly's test, and Greenhouse-Geisser corrections were applied when sphericity was violated (Mauchly's *P* < 0.05). The analyses focused on time × group interactions, main effects, and *post-hoc* comparisons. For significant interactions or main effects, Bonferroni-adjusted pairwise *t*-tests were conducted to evaluate within-group changes (pre- vs. postintervention). Results are reported as mean ± standard deviation (SD), with statistical significance set at *P* < 0.05. Effect sizes included partial $\eta_{p}^{2}$ (for ANOVA effects: $\eta_{p}^{2}$ ≥ 0.01 = small, ≥0.06 = medium, ≥0.14, large) and Cohen's *d* (Cohen, 1988) for within-group pre-post intervention effects (interpreted as *d* ≥ 0.20 = small, ≥0.50 = medium, ≥0.80 = large).

## 3 Results

### 3.1 Baseline characteristics

No significant differences were observed between the HIIT, Tabata, and MICT groups at baseline in terms of age, body weight, BMI, physical fitness, and cognitive ability (all *P* > 0.05; Table 1). The participants had a mean age of 19.07 ± 0.76 years. Scores on the comprehensive cognition questionnaire (CCQ) were comparable across the groups (HIIT: 58.93 ± 7.09; Tabata: 56.36 ± 8.59; MICT: 58.03 ± 8.80; *P* = 0.494).

### 3.2 Physical fitness

**Figure 5:**
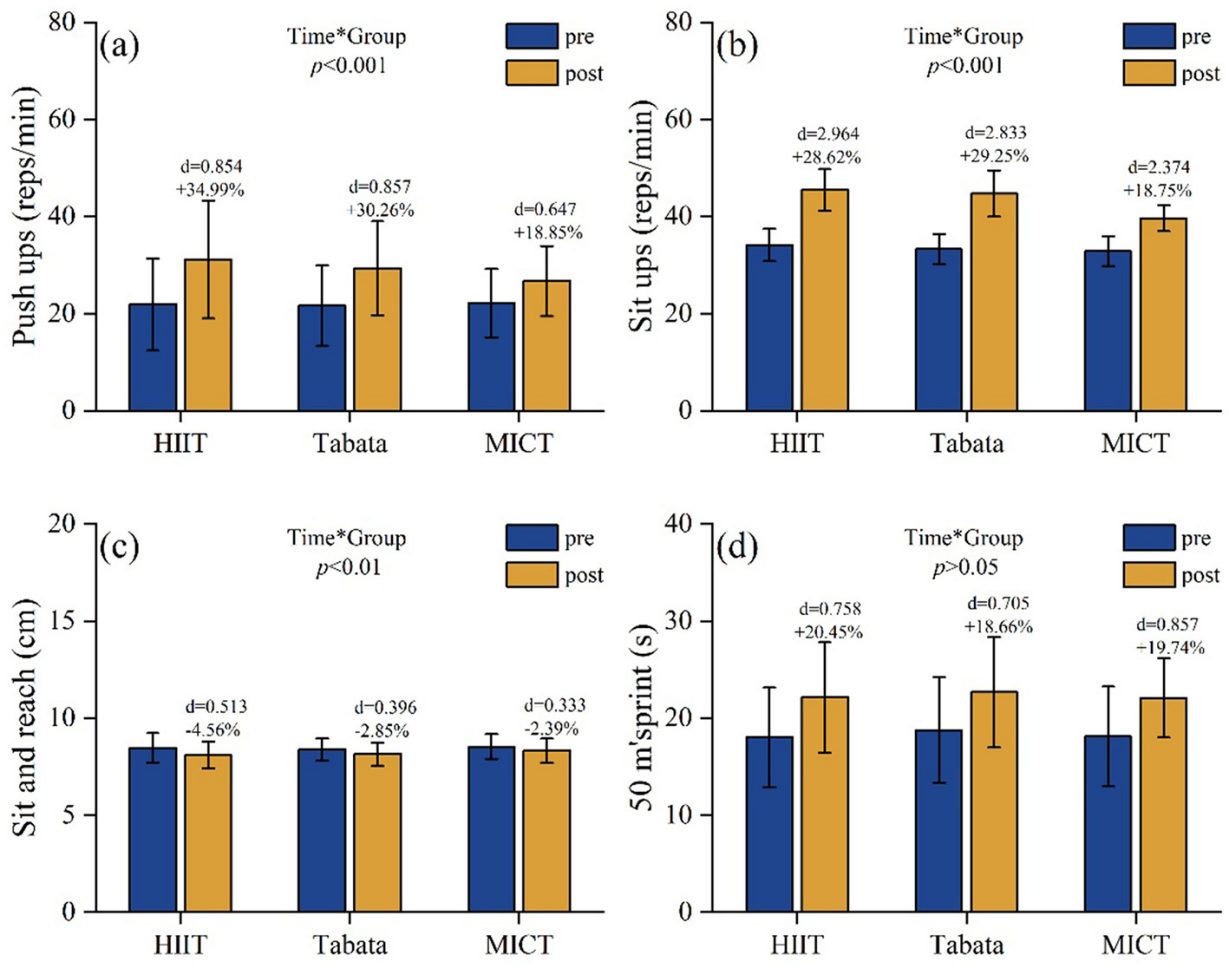
Pre/post-intervention changes in **(a)** push-ups (reps/min); **(b)** sit-ups (reps/min); **(c)** sit-and-reach (cm); and **(d)** 50-m sprint (s). Data: mean ± SD; error bars: 95% CI; Δ = change from baseline; effect of interaction: repeated-measures two-way ANOVA with Bonferroni correction.

Table 3 shows the results of the repeated-measures two-way ANOVA for the College students physical fitness and cognitive ability tests after the experimental intervention. Push-ups: a significant main effect of time [*F*(2,81) = 560.15, *P* < 0.001, $\eta_{p}^{2}$ = 0.874, large] indicated an overall improvement in all groups. No significant main effect of group [*F*(2,81) = 0.375, *P* = 0.678, $\eta_{p}^{2}$ = 0.009, trivial] was observed, suggesting no initial intergroup differences in the pre-test values. A significant time × group interaction [*F*(2,81) = 20.4, *P* < 0.001, $\eta_{p}^{2}$ = 0.335, large] suggested differential improvement. *Post-hoc* analysis showed that the HIIT-30S group achieved the greatest improvement (Δ +9.29 reps, +34.99%), significantly exceeding both Tabata (Δ +7.72 reps, +30.26%) and MICT (Δ +4.61 reps, +18.85%) groups. Between-group comparisons revealed no significant differences (*P* > 0.05; Figure 5a).

**Table 3:** Post-experiment test results for participants (mean ± standard deviation) and repeated measures two-way ANOVA outcomes.

**Tests**	**HIIT (*****N*** = **28)**			**Tabata (*****N*** = **28)**			**MICT (*****N*** = **28)**			**ANOVA** ***P*** **(**$\eta_{\text{p}}^{2}$**)**		
	**Results**	Δ**%**	**Cohen's** ***d*** **(95% CI)**	**Results**	Δ**%**	**Cohen's** ***d*** **(95% CI)**	**Results**	Δ**%**	**Cohen's** ***d*** **(95% CI)**	**Time**	**Group**	**Time** × **group**
BMI (kg/m^2^)	20.98 ± 2.29^***^	−6.37	0.587 (1.11–1.65)	21.49 ± 2.19^***^	−5.02	0.505 (1.02–1.19)	22.10 ± 2.52^***^	−3.47	0.317 (−0.68 to 0.88)	0.001 (0.863)	0.442 (0.021)	0.001 (0.242)
**Physical fitness**												
Push-ups	31.18 ± 12.1^***^	+35.0	0.854 (−10.75 to −7.84)	29.36 ± 9.69^***^	+30.3	0.857 (−8.84 to −6.59)	26.75 ± 7.21^***^	+18.9	0.647 (−5.02 to −4.19)	0.001 (0.874)	0.689 (0.009)	0.001 (0.335)
Sit-ups	45.5 ± 4.28^***^	+28.7	2.964 (−12.73 to −10.06)	44.75 ± 4.77^***^	+29.3	2.833 (−13.14 to −9.71)	39.64 ± 2.66^***^	+18.7	2.374 (−7.24 to −6.33)	0.001 (0.902)	0.001 (0.19)	0.001 (0.311)
50 m (s)	8.10 ± 0.68^***^	−4.56	0.513 (0.25–0.5)	8.15 ± 0.59^***^	−2.85	0.396 (0.02–0.19)	8.32 ± 0.62^***^	−2.39	0.333 (0.01–0.16)	0.001 (0.648)	0.588 (0.013)	0.004 (0.128)
SST (cm)	22.14 ± 5.71^***^	+20.5	0.758 (−4.57 to −3.65)	22.68 ± 5.68^***^	+18.7	0.705 (−4.35 to −3.5)	22.08 ± 4.08^***^	+19.7	0.857 (−4.67 to −3.29)	0.001 (0.896)	0.871 (0.003)	0.877 (0.003)
800 m (s)	207.43 ± 21.58^***^	−5.52	0.538 (9.46–14.11)	209.43 ± 22.89^***^	−3.88	0.389 (5.37–11.2)	213.14 ± 17.12^***^	−2.4	0.287 (4.15–6.21)	0.001 (0.689)	0.887 (0.003)	0.001 (0.185)
HRR (%)	2.88 ± 0.3^***^	+12.7	1.191 (−0.38 to −0.3)	2.84 ± 0.26^***^	+8.38	0.885 (−0.26 to −0.19)	2.74 ± 0.22^***^	+5.47	0.608 (−0.17 to −0.12)	0.001 (0.88)	0.734 (0.008)	0.001 (0.454)
**Cognitive ability**												
CCA	115.61 ± 3.14^***^	+7.8	3.009 (−9.14 to −8.22)	117.70 ± 3.31^***^	+9.3	3.325 (−11.03 to −9.9)	112.64 ± 2.85^***^	+3.57	1.477 (−4.72 to −3.39)	0.001 (0.967)	0.052 (0.071)	0.001 (0.779)
LNS	10.56 ± 0.99^***^	+17.0	1.763 (−1.83 to −1.48)	10.61 ± 0.72^***^	+17.2	2.133 (−1.85 to −1.48)	9.91 ± 0.76^***^	+9.71	1.174 (−0.99 to −0.84)	0.001 (0.937)	0.299 (0.029)	0.001 (0.48)
TMA	76.93 ± 4.00^***^	+14.7	2.638 (−11.83 to −9.17)	78.11 ± 3.88^***^	+15.2	2.77 (−12.15 to −9.78)	70.64 ± 3.58^***^	+5.51	1.116 (−4.3 to −3.28)	0.001 (0.906)	0.001 (0.185)	0.001 (0.594)
ERA	81.61 ± 4.92^***^	+19.7	3.082 (−15.69 to −13.6)	82.86 ± 5.17^***^	+22.6	3.311 (−18.3 to −15.27)	73.93 ± 5.33^***^	+13.7	2.351 (−10.56 to 8.37)	0.001 (0.95)	0.001 (0.223)	0.001 (0.491)
SRT (ms)	219.29 ± 18.13^***^	−12.8	1.607 (25.84–33.8)	213.96 ± 14.80^***^	−17.3	2.435 (35.09–46.13)	232.82 ± 14.49^***^	−6.2	0.896 (11.66–18.2)	0.001 (0.896)	0.285 (0.03)	0.001 (0.476)
CRT (ms)	334.04 ± 31.02^***^	−12.2	1.563 (35.51–51.49)	325.71 ± 35.72^***^	−18.2	1.991 (55.23–75.06)	351.79 ± 21.69^***^	−8.41	1.415 (27.6–34.12)	0.001 (0.854)	0.231 (0.036)	0.001 (0.352)
CT (ms)	453.18 ± 30.68^***^	−8.61	1.33 (35.95–45.62)	449.93 ± 29.94^***^	−10.8	1.766 (46.51–55.78)	471.86 ± 24.19^***^	−4.89	1.109 (17.08–30.28)	0.001 (0.874)	0.689 (0.009)	0.001 (0.335)

SST, seated stretch test; HRR, heart rate reserve; CCA, core cognitive ability; LNS, letter-number sequencing; TMA, time management assessment; ERA, emotional regulation ability; SRT, simple reaction time; CRT, choose reaction time; CT, continuous reaction time; Δ%, percentage change before and after the experiment; CI, confidence interval.

^*^Significant difference compared with the baseline test, *P* < 0.05, ^**^P < 0.01, ^***^P < 0.00.

Sit-ups: a significant main effect of time [*F*(2,81) = 747.75, *P* < 0.001, $\eta_{p}^{2}$ = 0.902 (large)] indicated an overall improvement in the number of sit-ups across all groups. A significant main effect of group [*F*(2,81) = 9.486, *P* < 0.001, $\eta_{p}^{2}$ = 0.19 (large)] revealed significant intergroup differences. A significant time × group interaction [*F*(2,81) = 18.25, *P* < 0.001, $\eta_{p}^{2}$ = 0.311 (large)] suggested a differential improvement. *Post-hoc* analysis showed that the HIIT-30S group achieved the greatest improvement (Δ +11.39 reps, +28.62%), significantly exceeding both Tabata (Δ +11.43 reps, +29.25%) and MICT (Δ +6.78 reps, +18.75%) groups. Between-group comparisons confirmed that HIIT-30S outperformed MICT [*P* < 0.001, *d* = 1.646 (large)] and Tabata [*P* < 0.001, *d* = 1.325 (large)]. No significant difference was observed between HIIT-30S and Tabata (*P* = 1.000; Figure 5b).

Sit-and-reach test: a significant main effect of time [*F*(2,81) = 697.55, *P* < 0.001, $\eta_{p}^{2}$ = 0.896 (large)] indicated an overall improvement in all groups. The main effects of group [*F*(2,81) = 0.139, *P* = 0.871, $\eta_{p}^{2}$ = 0.003 (trivial)] and time × group interaction [*F*(2,81) = 0.131, *P* = 0.877, $\eta_{p}^{2}$ = 0.003 (trivial)] were not significant, suggesting no differential effects. *Post-hoc* analysis showed that HIIT-30S achieved greater gains (Δ +4.11 cm, +20.45%) than MICT (Δ +3.98 cm, +19.74%) and Tabata (Δ +3.92 cm, +18.66%). Between-group comparisons revealed no significant differences (*P* > 0.05; Figure 5c).

50-m sprint: a significant main effect of time [*F*(2,81) = 149.41, *P* < 0.001, $\eta_{p}^{2}$ = 0.648 (large)] indicated an overall improvement. Main effects of group [*F*(2,81) = 0.534, *P* = 0.588, $\eta_{p}^{2}$ = 0.013 (small)] and time × group interaction [*F*(2,81) = 5.92, *P* = 0.004, $\eta_{p}^{2}$ = 0.128 (large)] were observed; the former suggested no significant intergroup differences, whereas the latter indicated differential changes in the same. *Post-hoc* analysis showed that HIIT-30S achieved the greatest improvement (Δ −0.37 s, −4.56%), surpassing MICT (Δ −0.21 s, −2.39%) and Tabata (Δ −0.23 s, −2.85%). Between-group comparisons showed no significant differences (*P* > 0.05; Figure 5d).

**Figure 6:**
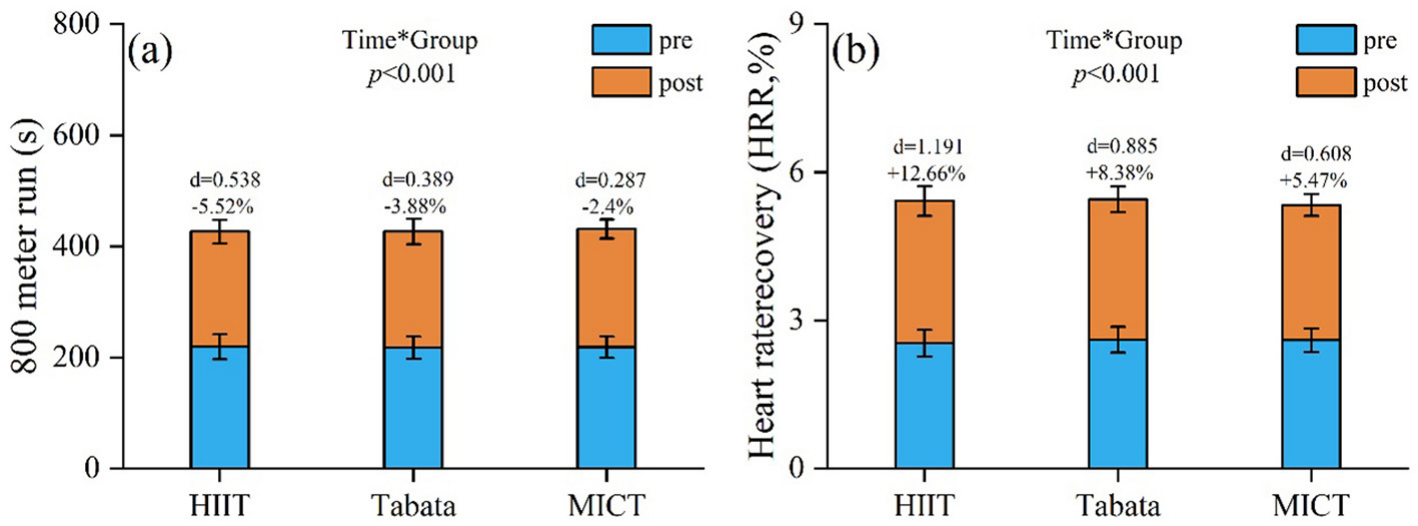
Pre/post-intervention changes in **(a)** 800-m run (s); **(b)** heart rate recovery (HRR, %). Data: mean ± SD; error bars: 95% CI; Δ = change from baseline; effect of interaction: repeated-measures two-way ANOVA with Bonferroni correction.

800-m run: a significant main effect of time [*F*(2,81) = 179.28, *P* < 0.001, $\eta_{p}^{2}$ = 0.689 (large)] indicated an overall improvement. Main effects of group [*F*(2,81) = 0.12, *P* = 0.887, $\eta_{p}^{2}$ = 0.003 (trivial)] and time × group interaction [*F*(2,81) = 9.22, *P* < 0.001, $\eta_{p}^{2}$ = 0.185 (large)] were observed; the former suggested no significant intergroup differences, whereas the latter indicated differential changes in the same. *Post-hoc* analysis showed that HIIT-30S achieved the greatest improvement (Δ −11.78 s, −5.52%), surpassing Tabata (Δ −8.28 s, −3.88%) and MICT (Δ −5.18 s, −2.4%). Between-group comparisons revealed no significant differences (*P* > 0.05; Figure 6a).

Heart rate recovery (HRR): a significant main effect of time [*F*(2,81) = 594.46, *P* < 0.001, $\eta_{p}^{2}$ = 0.88 (large)] indicated an overall improvement in HRR. Main effects of group [*F*(2,81) = 0.311, *P* = 0.734, $\eta_{p}^{2}$ = 0.008 (trivial)] and time × group interaction [*F*(2,81) = 33.68, *P* < 0.001, $\eta_{p}^{2}$ = 0.454 (large)] were observed; the former suggested no significant intergroup differences, whereas the latter indicated differential changes in the same. *Post-hoc* analysis showed that HIIT-30S achieved the greatest improvement (Δ +12.66%), surpassing Tabata (Δ +8.38%) and MICT (Δ +5.47%). Between-group comparisons revealed no significant differences (*P* > 0.05; Figure 6b).

### 3.3 Cognitive ability

**Figure 7:**
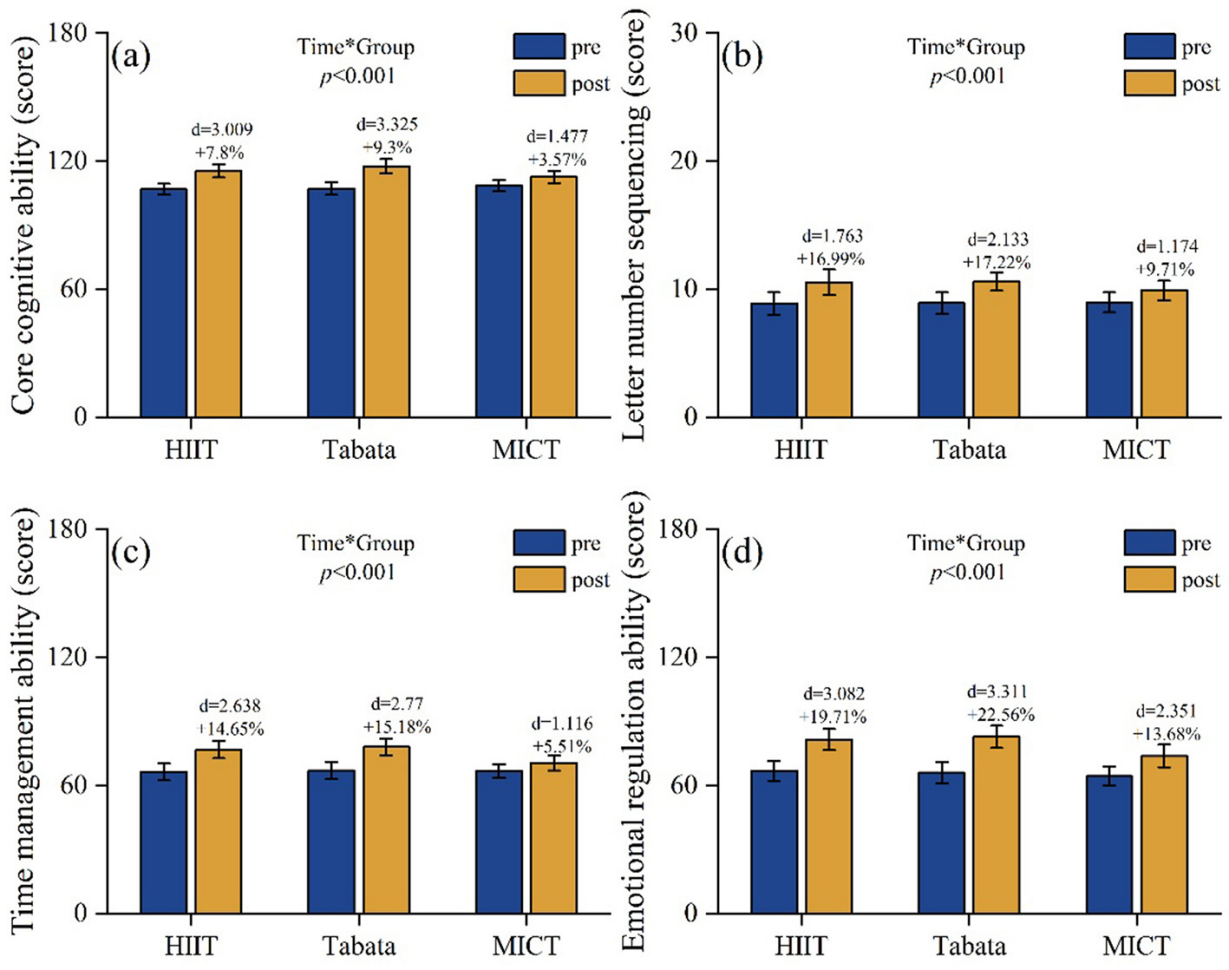
Pre/post-intervention changes in **(a)** core cognitive ability (WAIS-IV score); **(b)** letter-number sequencing (score); **(c)** time management ability (score); and **(d)** emotional regulation ability (score). Data: mean ± SD; error bars: 95% CI; Δ = change from baseline; effect of interaction: repeated-measures two-way ANOVA with Bonferroni correction.

WAIS core cognition: a significant main effect of time [*F*(2,81) = 2,341.32, *P* < 0.001, $\eta_{p}^{2}$ = 0.967 (large)] indicated an overall improvement. Main effects of group [*F*(2,81) = 3.08, *P* = 0.052, $\eta_{p}^{2}$ = 0.071 (medium)] and time × group interaction [*F*(2,81) = 142.89, *P* < 0.001, $\eta_{p}^{2}$ = 0.779 (large)] were observed; the former approached significance (*P* = 0.052), whereas the latter indicated differential changes in the results. *Post-hoc* analysis showed that the Tabata group achieved the greatest improvement (Δ +10.47 points, +9.3%), surpassing HIIT-30S (Δ +8.68 points, +7.8%) and MICT groups (Δ +4.05 points, +3.57%). Between-group comparisons confirmed that the Tabata group improved significantly more than the HIIT-30S [*P* = 0.019, *d* = 0.648 (medium)] and MICT [*P* < 0.001, *d* = 1.638 (large)] groups, whereas the HIIT-30S group showed greater improvement than the MICT group [*P* < 0.001, *d* = 0.99 (large); Figure 7a].

Letter-number sequencing test: a significant main effect of time [*F*(2,81) = 1,200.71, *P* < 0.001, $\eta_{p}^{2}$ = 0.937 (large)] indicated an overall improvement. Main effects of group [*F*(2,81) = 1.225, *P* = 0.299, $\eta_{p}^{2}$ = 0.029 (small)] and time × group interaction [*F*(2,81) = 37.4, *P* < 0.001, $\eta_{p}^{2}$ = 0.48 (large)] were observed; the former showed no significant intergroup differences, whereas the latter indicated differential changes in the same. *Post-hoc* analysis showed that the Tabata group achieved the greatest improvement (Δ +1.68 points, +17.22%), surpassing the HIIT-30S (Δ +1.66 points, +16.99%) and MICT groups (Δ +0.91 points, +9.71%). Between-group comparisons confirmed that Tabata training improved significantly more than MICT [*P* = 0.007, *d* = 0.946 (large)] and HIIT-30S improved significantly more than MICT [*P* = 0.015, *d* = 0.737 (medium)]. No significant difference was found between HIIT-30S and Tabata (*P* = 1.000; Figure 7b).

Time management ability: a significant main effect of time [*F*(2,81) = 781.6, *P* < 0.001, $\eta_{p}^{2}$ = 0.906 (large)] indicated an overall improvement in time management ability. A significant main effect of group [*F*(2,81) = 9.19, *P* < 0.001, $\eta_{p}^{2}$ = 0.185 (large)] suggested significant intergroup differences in the results. A significant time × group interaction [*F*(2,81) = 59.35, *P* < 0.001, $\eta_{p}^{2}$ = 0.594 (large)] indicated differential changes over time. *Post-hoc* analysis showed that the Tabata group achieved the greatest improvement (Δ +10.97 points, +15.18%), surpassing the HIIT-30S (Δ +10.05 points, +14.65%) and MICT groups (Δ +3.78 points, +5.51%). Between-group comparisons confirmed significant improvements for Tabata vs. MICT [*P* < 0.001, *d* = 2.000 (large)] and HIIT-30S vs. MICT [*P* < 0.001, *d* = 1.657 (large)], with no significant difference between HIIT-30S and Tabata (*P* = 0.757; Figure 7c).

Emotional management ability: a significant main effect of time [*F*(2,81) = 1,534.02, *P* < 0.001, $\eta_{p}^{2}$ = 0.95 (large)] indicated an overall improvement in emotional management ability. A significant main effect of group [*F*(2,81) = 11.66, *P* < 0.001, $\eta_{p}^{2}$ = 0.223 (large)] suggested significant intergroup differences were present. A significant time × group interaction [*F*(2,81) = 38.99, *P* < 0.001, $\eta_{p}^{2}$ = 0.491 (large)] indicated differential changes over time. *Post-hoc* analysis showed that the Tabata group achieved the greatest improvement (Δ +16.79 points, +22.56%), surpassing the HIIT-30S (Δ +14.68 points, +19.71%) and MICT (Δ +9.47 points, +13.68%). Between-group comparisons confirmed significant improvements for Tabata vs. MICT [*P* < 0.001, *d* = 1.700 (large)] and HIIT-30S vs. MICT [*P* < 0.001, *d* = 1.497 (large)], with no significant difference between HIIT-30S and Tabata (*P* = 1.000; Figure 7d).

**Figure 8:**
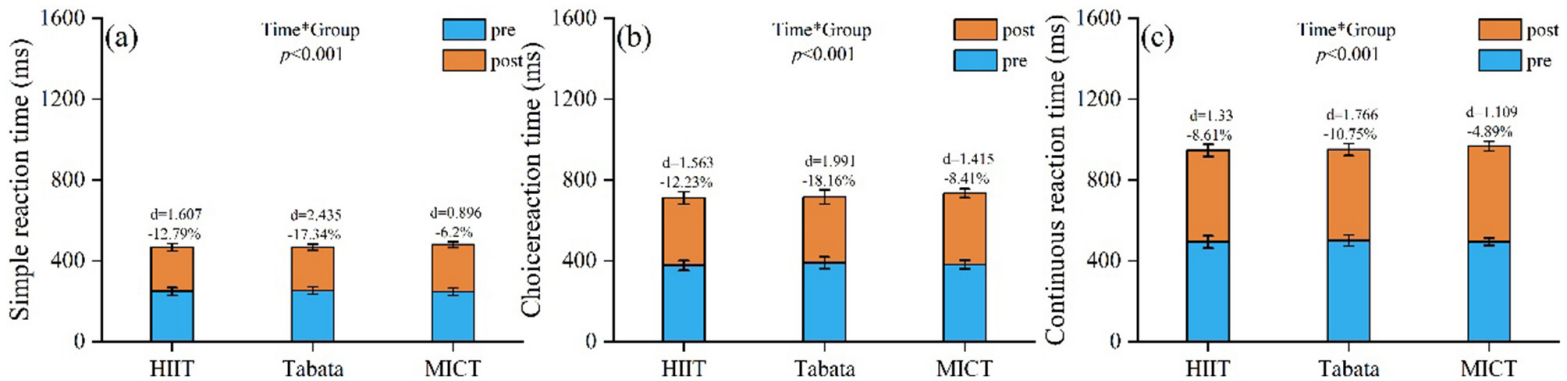
Pre/post-intervention changes in **(a)** simple reaction time (ms); **(b)** choice reaction time (ms); **(c)** continuous reaction time (ms). Data: mean ± SD; error bars: 95% CI; Δ = change from baseline; effect of interaction: repeated-measures two-way ANOVA with Bonferroni correction.

Simple reaction time: a significant main effect of time [*F*(2,81) = 538.29, *P* < 0.001, $\eta_{p}^{2}$ = 0.869 (large)] indicated an overall improvement. No significant main effect of group [*F*(2,81) = 1.27, *P* = 0.285, $\eta_{p}^{2}$ = 0.03 (small)] was found. A significant time × group interaction [*F*(2,81) = 36.85, *P* < 0.001, $\eta_{p}^{2}$ = 0.476 (large)] suggested differential changes over time. *Post-hoc* analysis showed that the Tabata group achieved the greatest improvement (Δ −40.61 ms, −17.34%), surpassing the MICT (Δ −14.93 ms, −6.2%) and HIIT-30S groups (Δ −29.82 ms, −12.79%). Between-group comparisons confirmed significant improvements for Tabata vs. MICT [*P* < 0.001, *d* = 1.288 (large)] and HIIT-30S vs. MICT [*P* = 0.006, *d* = 0.824 (large)]. No significant difference was found between HIIT-30S and Tabata (*P* = 0.641; Figure 8a).

Choice reaction time: a significant main effect of time [*F*(2,81) = 473.97, *P* < 0.001, $\eta_{p}^{2}$ = 0.854 (large)] indicated an overall improvement. No significant main effect of group [*F*(2,81) = 1.49, *P* = 0.231, $\eta_{p}^{2}$ = 0.036 (small)] was found. A significant time × group interaction [*F*(2,81) = 21.97, *P* < 0.001, $\eta_{p}^{2}$ = 0.352 (large)] suggested differential changes over time. *Post-hoc* analysis showed that the Tabata group achieved the greatest improvement (Δ −65.15 ms, −18.16%), surpassing the HIIT-30S (Δ −43.5 ms, −12.23%) and MICT groups (Δ −30.85 ms, −8.41%). Between-group comparisons confirmed a significant Tabata vs. MICT improvement [*P* = 0.005, *d* = 0.883 (large)]. No significant differences were found between HIIT-30S and Tabata (*P* = 0.909) or between HIIT-30S and MICT (*P* = 0.09; Figure 8b).

Continuous reaction time: a significant main effect of time [*F*(2,81) = 636.1, *P* < 0.001, $\eta_{p}^{2}$ = 0.887 (large)] indicated an overall improvement. No significant main effect of group [*F*(2,81) = 1.163, *P* = 0.318, $\eta_{p}^{2}$ = 0.028 (small)] was observed. A significant time × group interaction [*F*(2,81) = 27.467, *P* < 0.001, $\eta_{p}^{2}$ = 0.404 (large)] suggested differential changes over time. *Post-hoc* analysis showed that the Tabata group achieved the greatest improvement (Δ −51.14 ms, −10.75%), surpassing the MICT (Δ −23.68 ms, −4.89%) and HIIT-30S groups (Δ −40.78 ms, −8.61%). Between-group comparisons confirmed significant improvements for HIIT-30S vs. MICT [*P* = 0.048, *d* = 0.676 (medium)] and Tabata vs. MICT [*P* = 0.015, *d* = 0.806 (large)]. No significant difference was found between HIIT-30S and Tabata (*P* = 1.000; Figure 8c).

### 3.4 Comprehensive cognition questionnaire (CCQ)

At 8 weeks, a significant main effect of time [*F*(2,81) = 1,417.5, *P* < 0.001, $\eta_{p}^{2}$ = 0.946 (large)] indicated an overall improvement across groups. A significant main effect of group [*F*(2,81) = 3.44, *P* = 0.037, $\eta_{p}^{2}$ = 0.078 (medium)] suggested intergroup differences. A significant time × group interaction [*F*(2,81) = 14.5, *P* < 0.001, $\eta_{p}^{2}$ = 0.264 (large)] indicated differential changes over time. *Post-hoc* analysis showed that the Tabata group outperformed the HIIT-30S (score 84.43 ± 4.41, Δ +28.7 points, +39.88%) and MICT groups (score 77.89 ± 6.14, Δ +19.86 points, +29.22%). HIIT-30S showed greater improvement than MICT [*P* < 0.001, *d* = 1.533 (large)], and Tabata showed greater improvement than MICT [*P* < 0.001, *d* = 1.223 (large)]. No significant difference was found between HIIT-30S and Tabata (*P* = 1.000).

By 8–12 weeks: a significant main effect of time [*F*(2,81) = 19.801, *P* < 0.001, $\eta_{p}^{2}$ = 0.196 (large)] indicated overall improvement. A significant main effect of group [*F*(2,81) = 31.266, *P* < 0.001, $\eta_{p}^{2}$ = 0.436 (large)] suggested intergroup differences. No significant time × group interaction [*F*(2,81) = 1.15, *P* = 0.322, $\eta_{p}^{2}$ = 0.028 (small)] was observed, indicating no differential changes over time. *Post-hoc* analysis revealed that HIIT-30S maintained gains (+3.05%, *P* < 0.001, *d* = 0.88 (large)], Tabata showed smaller improvements (+1.97%, *P* = 0.025, *d* = 0.46 (small)], and MICT showed nonsignificant changes (+1.46%, *P* = 0.181). HIIT-30S showed greater improvement than MICT [*P* < 0.001, *d* = 1.77 (large)] and Tabata [*P* < 0.001, *d* = 1.43 (large)]. No significant difference was found between the HIIT-30S and Tabata groups (*P* = 0.351).

## 4 Discussion

This randomized controlled trial investigated the differential effects of HIIT and Tabata on physical fitness and cognitive ability in sedentary college students. Key findings include the following: (1) both interventions significantly improved physical fitness and cognitive ability compared with the baseline; (2) the HIIT-30S protocol yielded greater improvements in cardiovascular endurance and muscle strength; (3) in contrast, the Tabata protocol demonstrated more pronounced cognitive benefits than the HIIT one; and (4) both protocols resulted in significant cognitive enhancement throughout the 12-week follow-up period. These results contribute to the understanding of exercise prescription by highlighting the protocol-specific advantages tailored to distinct health objectives.

Our findings demonstrate that the HIIT-30S protocol induced greater improvements in cardiovascular endurance, upper-body muscular endurance, and lower-body explosive power than the Tabata protocol did. These results are consistent with established physiological principles of HIIT. The prolonged high-intensity work periods (30 s at ≥85% HRmax) in HIIT-30S are likely pivotal for driving significant physiological adaptations (Mosley, 2014). Enhanced cardiovascular efficiency, potentially involving cardiac remodeling (Mahjoub et al., 2019), leads to increased stroke volume and oxygen transport capacity, directly supporting sustained high-intensity activity and contributing significantly to the observed endurance gains (Liu and Li, 2024). Concurrently, the high mechanical tension and metabolic stress resulting from exercises targeting specific muscle groups (e.g., push-ups and burpees) stimulate key neuromuscular adaptations, such as enhanced motor unit recruitment (Gavanda et al., 2022; Cannon and Cafarelli, 1987), which likely underpins the improvements in both muscular endurance and explosive power observed with HIIT-30S. The intense metabolic demands during HIIT-30S may stimulate mitochondrial biogenesis via pathways such as AMPK-PGC-1α (Shao et al., 2023a), enhancing cellular energy production capacity. Furthermore, adaptations within the respiratory system, including increased ventilatory efficiency, are expected to contribute to enhanced cardiovascular endurance (Sheel and Romer, 2012). The duration of our 8-week study may not fully capture long-term physiological adaptations, and our single sample size limits the generalizability of the findings. Individual responses to HIIT protocols can vary considerably, and our assessment focused on the specific performance metrics of the participants. Future research could benefit from longer intervention periods to assess chronic adaptations and more comprehensive physiological parameters. In conclusion, the superior physiological adaptations observed with HIIT-30S likely stem from a synergistic combination of cardiovascular improvements and enhanced neuromuscular coordination. This makes HIIT-30S a potent and potentially time-efficient strategy for enhancing key performance metrics relevant to athletic development and active living, particularly in populations with limited time training.

Our findings elucidate protocol-specific patterns of cognitive enhancement following High-Intensity Interval Training, highlighting a potential dose-response relationship with training intensity and structure. Notably, the Tabata protocol elicited superior improvements compared with the HIIT-30S protocol, particularly in core cognitive abilities and working memory. These results resonate with models proposing that structured interval timing, coupled with repetitive metabolic stress, facilitates neuroplasticity related to prefrontal-hippocampal circuit optimization (Reyes-Amigo et al., 2022). The precise temporal structure of the Tabata protocol's rigid 20:10-second work-rest ratio may enhance cognitive predictability, thereby facilitating sustained attentional resource allocation and optimizing executive function (Shao et al., 2023b). Furthermore, consistent high-intensity bursts may drive specific adaptations within the prefrontal cortex and hippocampus relevant to these cognitive domains (Andrews et al., 2020). In contrast, although HIIT also improved cognitive measures, its pattern of enhancement was less pronounced in these specific domains than in Tabata. Moreover, the Tabata protocol showed greater improvements in Emotional Regulation and Time Management than HIIT-30S. The unique combination of intense acute metabolic stress and subsequent recovery inherent in the Tabata protocol may promote specific neuroplastic changes relevant to cognitive control (Mosley, 2014). This finding aligns with existing research, which indicates that HIIT can decrease cardiac sympathovagal tone in university students (May et al., 2019). While both protocols demonstrated cognitive gains, the differential pattern suggests distinct underlying mechanisms driven by unique and acute physiological demands. Compared with the HIIT-30S protocol, the Tabata protocol could potentially enhance the plasticity of brain regions involved in executive functioning, emotional processing, and self-regulation, such as the prefrontal and anterior cingulate cortices (Reyes-Amigo et al., 2022). This neuroplasticity may underlie the observed improvements in the management of emotional responses and time allocations. A critical consideration regarding the cognitive findings, particularly those involving Emotional Regulation, pertains to the reliance on self-reported measures (ERQ). Self-report instruments are susceptible to potential social desirability bias or recall inaccuracies, which can influence perceptions of emotional state changes. This methodological choice contrasts with studies employing objective autonomic indices, which, while offering a different perspective, sometimes report weaker or more variable exercise-emotion regulation correlations (Palefsky, 2022). This discrepancy underscores the need for multimodal assessment frameworks that incorporate both subjective and objective measures in exercise cognition research. Such integrated approaches are crucial, particularly given the potential limitations of self-reports in capturing the nuances of transient physiological changes associated with acute exercise and the complex interplay between affect and cognition.

Our findings demonstrate that reaction times significantly decreased across all groups, with the magnitude of improvement being greater in the Tabata group for both simple and choice reaction time tasks. Simple and choice reaction time reduction suggests optimization within the cerebellar-thalamocortical pathways related to rapid motor pattern alternation (Habas et al., 2019), while comparable continuous reaction time improvements may indicate engagement and optimization of the shared attention network (Lawrence et al., 2003). Nevertheless, the absence of concurrent neuroimaging data (e.g., fMRI) precludes direct comparison with fMRI that studies demonstrate intensity-dependent prefrontal activation patterns during cognitive tasks. Moreover, behavioral metrics alone cannot fully distinguish between genuine improvements in neural efficiency and compensatory strategies, highlighting the necessity of complementary neurophysiological and neuroimaging techniques to fully elucidate the underlying mechanisms of exercise-induced cognitive benefits.

Longitudinal cognitive assessments over 12 weeks revealed significant improvements in the HIIT-30S and Tabata groups, whereas no significant changes were observed in the MICT group. Longitudinal data have revealed sustained cognitive benefits of HIIT, which may be mediated by moderate-intensity-induced BDNF elevation, promoting sustained neuroplasticity beyond the acute session (Cobianchi et al., 2017). This contrasts with the potentially more transient cognitive effects associated with Tabata's acute 90% HRmax stimuli, which may prioritize immediate cerebrovascular adaptations (e.g., blood flow increases) over downstream, sustained neurotrophic effects, such as BDNF synthesis. Notably, our finding of greater cognitive sustainability with HIIT contrasts with that of Mekari et al. (2020), who observed comparable cognitive sustainability between HIIT and moderate-intensity continuous training (MIC) in older adults. This discrepancy likely reflects age-dependent neuroplasticity mechanisms. While older adults may exhibit neuroplastic responses optimized for maintaining function, the collegiate population studied here likely possesses a different neuroplastic profile characterized by potentially greater baseline neural efficiency, which may dampen the magnitude of protocol-specific cognitive adaptations observed over 12 weeks. This highlights that cognitive responses to interval training may be more pronounced or persistent in populations with potentially greater plasticity or different baseline neural requirements. Finally, the absence of biomarker assays [e.g., serum brain-derived neurotrophic factor (BDNF) and functional magnetic resonance imaging (FMRI)] limits the causal interpretation of these neurophysiological mechanisms. The dissociation between HIIT's superior physical fitness improvements of HIIT (e.g., push-ups, sit-ups, and 50-m sprint) and attenuated cognitive gains supports fatigue-mediated interference models. HIIT's elevated exertion (RPE: 7.11 ± 1.4 vs. Tabata's 6.55 ± 1.11) may transiently disrupt glutamatergic homeostasis in working memory networks, as evidenced by its smaller cognitive ability improvements. This contrasts with the findings of Hatch et al. (2021), who reported proportional physical-cognitive HIIT gains in adolescents, likely due to their longer session duration (30 vs. 60 min) and lower baseline cognitive loads.

## 5 Conclusion

This randomized controlled trial compared the effects of HIIT-30S and Tabata protocols on physical fitness and cognitive function in sedentary college students. Both interventions significantly improved physical and cognitive performances. These enhancements may be attributed to the relatively high neuroplasticity observed in college students, combined with the specific demands of the training protocols, which promoted adaptations within a short period (8 weeks). HIIT-30S demonstrated greater improvements in cardiorespiratory endurance, muscular strength, and speed, potentially linked to its longer work and rest intervals, fostering enhanced energy metabolic system efficiency, and neuromuscular adaptation. Conversely, the structured timing of the Tabata protocol contributed to greater cognitive gains and was preferred by the participants. Notably, while Tabata initially yielded a more pronounced cognitive enhancement than HIIT-30S at 8 weeks, the 12-week follow-up revealed sustained cognitive benefits in both groups, with HIIT-30S eventually showing superior cognitive performance compared to Tabata. These findings underscore that the work-to-rest ratio is a key determinant of physiological and neural adaptations, tailoring training protocols to specific physical fitness or cognitive goals of the individual. The limitations of this study include the relatively small and homogeneous sample size (*N* = 28, all from one university), the short intervention duration (8 weeks), the lack of a passive control group, and the reliance on self-reported measures for certain psychological constructs (e.g., emotional regulation using the ERQ and time management ability using TMA), which are susceptible to social desirability bias or recall inaccuracies. Furthermore, key physiological parameters, such as VO_2_max, anaerobic power, and neurophysiological indicators, were not measured using precise instruments. Additionally, no statistical adjustments were made for potential confounding variables such as sleep quality and duration, nutritional habits, academic stress levels, or other lifestyle factors. Future research should integrate biological markers (e.g., fMRI, BDNF), explore the synergistic effects of novel exercise protocols (e.g., HIIT-Tabata alternation), develop efficient micro-interventions (e.g., 5-min interclass exercise “snacks”), and conduct long-term tracking to elucidate the mechanisms by which exercise promotes physical and cognitive health in sedentary college students while optimizing intervention strategies. Consider controlling for or including sleep quality and duration, nutritional habits, academic stress levels, or other lifestyle factors in the statistical models (e.g., ANCOVA or mixed-effects models).

## Data Availability

The raw data supporting the conclusions of this article will be made available by the authors, without undue reservation.
