# Correction: HIIT and Tabata protocols for improving physical and cognitive health in sedentary college students: a randomized trial

**DOI:** 10.3389/fpsyg.2026.1845030

**Published:** 2026-05-05

**Authors:** Yadong Xue, Ning Xu, Meng Zhang

**Affiliations:** 1 School of Physical Education, Yan'an University, Yan'an, Shaanxi, China; 2 School of Physical Education, Guangzhou Huali College, Guangzhou, China; 3 School of Exercise and Health, Shanghai University of Sport, Shanghai, China

**Keywords:** cognitive function enhancement, high-intensity interval training, physical fitness, sedentary college students, Tabata protocol

In the published article, there were errors in the author affiliations. Affiliation 3 School of Exercise and Health, Shanghai University of Sport, Shanghai, China was omitted for the author Meng Zhan. This affiliation has now been added for author Meng Zhang.

The author Ning Xu was incorrectly assigned to affiliation 1 School of Physical Education, Yan'an University, Yan'an, Shaanxi, China. This affiliation has now been removed for author Ning Xu.

In addition, affiliation 2 School of Physical Education, Guangzhou Huali College, Guangzhou, China was omitted from the publication. This affiliation has now been added for author Ning Xu.

The equal contribution statement was erroneously missing from authors Yadong Xue and Ning Xu. The correct statement is as follows:

Yadong Xue^1*†^, Ning Xu^2†^ and Meng Zhang^3^

^†^These authors have contributed equally to this work and share first authorship.

The original version of this article has been updated.

