# Correction: *N*-acyl-homoserine lactone-based quorum sensing beyond canonical lineages: insights from Actinomycetota

**DOI:** 10.3389/fmicb.2026.1861688

**Published:** 2026-05-08

**Authors:** Frontiers Production Office

**Affiliations:** Frontiers Media SA, Lausanne, Switzerland

**Keywords:** Actinomycetota, *Bradyrhizobium*, *Brenneria*, *N*-acyl-homoserine lactones, *Pseudonocardia*, quorum sensing, *Rhodococcus*

In the abstract, several scientific terms were not formatted correctly. The corrected abstract appears below:

Classically, quorum sensing (QS) is defined as a form of bacterial communication that regulates gene expression in response to population density and other environmental conditions. While a variety of QS systems exist, *N*-acyl homoserine lactone-based quorum sensing (AHL-QS) is the best characterized in Gram-negative Pseudomonadota (formerly Proteobacteria), often in medically relevant taxa. However, it remains unclear what AHL-QS systems look like in underexplored environments, and how often or far the canonical model extends beyond the Pseudomonadota. Here, we investigated AHL production in environmental bacteria from underexplored environments, including two newly described Pseudomonadota species, *Brenneria uluponensis* K61^T^, from a taro lo‘i in Hawai‘i, and *Bradyrhizobium prioriisuperbiae* BL16A^T^, from a lava cave on the Island of Hawai‘i, and two Gram-positive Actinomycetota (formerly Actinobacteria), *Pseudonocardia alni* GV4, from a culture of *Gloeobacter violaceus*, and *Rhodococcus kroppenstedtii* Y88A, from a dehydrated hypersaline mat on San Salvador Island, Bahamas. Custom hidden Markov models (HMMs) were used to identify putative *luxI/R* homologs, followed by analyses of genomic context, LuxI/R-family protein phylogeny, and LuxR domain architecture. AHL production was assessed using liquid chromatography-multiple reaction monitoring-mass spectrometry (LC-MRM-MS), and targeted gene disruption was performed in *R. kroppenstedtii* Y88A. Putative *luxI/R* homologs were identified in all strains. In *R. kroppenstedtii* Y88A, disruption of the sole *luxI* homolog resulted in loss of all detectable AHLs, indicating that this gene (designated *rhdI*) is required for AHL production under the tested conditions. Across Actinomycetota genomes, *luxI* genes occurred as solitary elements rather than canonical *luxI/R* pairs and were associated with genes linked to metabolism and redox processes. Phylogenetic analyses revealed that Actinomycetota LuxI/R-family proteins diverge from canonical systems, with LuxR-family proteins lacking canonical autoinducer-binding domains and instead comprising only helix-turn-helix regulatory architectures. Together, these findings expand the known diversity of AHL-QS systems into underexplored microbial lineages and environments and suggest broader ecological roles for AHL signaling. The canonical *luxI/R* model derived largely from Pseudomonadota may represent only one of several evolutionary architectures for AHL signaling, raising the possibility that AHL production in Actinomycetota operates through regulatory frameworks distinct from classical AHL-QS systems.

